# Reviews

**Published:** 1881-09

**Authors:** 


### Reviews.

**Supplement to *Ziemssen's* Cyclopædia of the Practice of Medicine.** Edited by GEORGE L PEABODY, M. D. New York: William Wood & Co., 27 Great Jones street. 1881.

This supplement is offered to the profession with the object to remove from this extensive work the few traces of time that the last few years have produced. It is the aim to give a concise account of the progress made in the various departments of medicine during the time that has elapsed since the several volumes of the cyclopædia were published, in other words to bring the work down to the present time. With this object in view, the publishers have called to the task many of our ablest medical writers, and have spared no expense, to offer in the original work, as well as in this supplement, the most complete library of practical medicine ever offered to the profession. While we may justly regard some of the volumes as too ponderous for ready reference, yet taken as a whole, we believe the cyclopædia to be a valuable and, indeed, an indispensible addition to a medical library. The profession may well second the efforts of the publishers in securing this volume to complete the work.

---

**Atlas of Skin Diseases.** By LOUIS A. DUHRING, M. D. Part IX. Philadelphia: J. B. Lippincott & Co. 1881.

Part IX concludes the series of valuable plates offered to the profession by Prof. Duhring. It contains plates illustrating eczema (rubrum), pemphigus, and ecthyma, with explanatory text furnishing a clinical lecture of the case, illustrated in the plate. The series, therefore, is a practical treatise on skin diseases, giving typical cases, with explanatory notes to each case, and plates of the highest artistic finish, which are an invaluable guide in the study of this otherwise difficult and obscure department. The author has added to his already enviable reputation in the work which he has thus given to the profession.

**Clinical Lectures on the Diseases of Old Age.** By J. M. CHARCOT, M.D., Professor in the Faculty of Paris, etc., etc. Translated by Leigh H. Hunt, B. Sc. M. D. With additional Lectures by ALFRED L. LOOMIS, M. D. New York: William Wood & Co., 27 Great Jones street. 1881.

**A Treatise on The Continued Fevers.** By JAMES C. WILSON, M. D., Lecturer on Physical Diagnosis at the Jefferson Medical College, Philadelphia, etc. With an introduction by J. M. DACOSTA, M. D. New York: Wm. Wood & Co., 27 Great Jones street.

These works belong to the library of Standard Medical Authors, issued by Messrs. Wood & Co., and demonstrate the wise choice of the enterprising publishers, not only in the subjects treated, but in the selection of authors.

The work of Prof. Charcot, to which Prof. Loomis has added valuable lectures on the Diseases of Old Age, is a most valuable addition to our medical literature, inasmuch as in no other work have we found a selection of subjects so admirably adapted to the wants of the profession.

The Treatise on Continued Fevers by Dr. Wilson, gives a clear and forcible exposition of the subjects of which it treats, dealing with the practical rather than with the theoretical, and placing before the profession a valuable guide in the study and treatment of a class of diseases, which are only too common.

We commend these works to the profession for their practical worth, and believe they will be regarded as an important addition to the library of the practitioner.

---

**Coulson on the Diseases of the Bladder and Prostate Gland.** Sixth edition. Revised by WALTER J. COULSON, F. R. C. S., Surgeon to St. Peter's Hospital for Stone, etc. New York: William Wood & Co. 1881.

We acknowledge that we never saw a copy of this book before, but if the fifth edition, which appeared a quarter of a century ago, exhausted the subject as perfectly as this does, we have no doubt that our fathers read it with as much pleasure and advantage as we expect to do. Necessarily the book has been almost wholly rewritten and new chapters added. Especially we mention the chapter about the causes of stone in the bladder, and the chapter about Bigelow's operation, litholapaxy, a method which rapidly has gained the acknowledgement of the profession, and may be considered as one of the greatest discoveries surgery has made in our days. The work will be found especially useful as a book of reference, and its moderate price as one of "Wood's Library of Standard Medical Authors," combined with its excellence, ought to ensure a large sale.

---

**A Practical Treatise on Impotence, Sterility and Allied Disorders of the Male Sexual Organs.** By SAMUEL W. GROSS, M. D., Lecturer on Venereal and Genito-Urinary Diseases in the Jefferson Medical College, etc. With sixteen illustrations. Philadelphia: Henry C. Lea's, Son & Co. 1881.

The importence of this subject cannot be overestimated, the more, as, according to the author, in sterile marriages the husband is at fault one instance in every six. The author maintains that these affections are not dependent, as generally has been believed, upon functional disorders of the testicles, but upon reflex disturbances of the genito-spinal centre, induced by lesions of the prostatic portion of the urethra. The book is divided into four chapters—impotence, sterility, spermatorrhœa, and prostatorrhœa, and is a very valuable addition to the scanty literature on this subject. A great many young men suffer from these disorders, and often, or generally, fall into the hands of quacks, because physicians make slight of their complaints. A more thorough knowledge of the profession on this subject is greatly desired and would, in a great measure, tend to abate quackery in its most offensive form.

---

**The Compend of Anatomy; for Use in the Dissecting Room and in Preparing for Examinations.** By JOHN B. ROBERTS, M. D., etc. Second edition. Philadelphia: C. C. Roberts & Co., 1118 Arch street. 1881.

That the second edition of this little work has appeared, is proof enough that it has found the favor with the students which it deserves. Older members of the profession would find it a convenient little book, by aid of which to recapitulate the anatomy.

